# Draft Genome Sequence of the Brazilian Toxic Bloom-Forming Cyanobacterium *Microcystis aeruginosa* Strain SPC777

**DOI:** 10.1128/genomeA.00547-13

**Published:** 2013-08-01

**Authors:** Marli F. Fiore, Danillo O. Alvarenga, Alessandro M. Varani, Caroline Hoff-Risseti, Elaine Crespim, Rommel T. J. Ramos, Artur Silva, Patricia D. C. Schaker, Karina Heck, Janaina Rigonato, Maria Paula C. Schneider

**Affiliations:** University of São Paulo, Center for Nuclear Energy in Agriculture, Piracicaba, São Paulo, Brazila; Faculdade de Ciências Agrárias e Veterinárias, Universidade Estadual Paulista, Campus de Jaboticabal, Departamento de Tecnologia, Jaboticabal, São Paulo, Brazilb; Federal University of Pará, Department of Genetics, Belém, Pará, Brazilc

## Abstract

*Microcystis aeruginosa* strain SPC777 is an important toxin-producing cyanobacterium, isolated from a water bloom of the Billings reservoir (São Paulo State, Brazil). Here, we report the draft genome sequence and initial findings from a preliminary analysis of strain SPC777, including several gene clusters involved in nonribosomal and ribosomal synthesis of secondary metabolites.

## GENOME ANNOUNCEMENT

*Microcystis aeruginosa* is a well-known bloom-forming freshwater species of cyanobacterium causing economical and ecological problems worldwide (1). Several strains of *M. aeruginosa* synthesize powerful toxins that represent a potentially serious threat for water quality, other aquatic organisms, and human health (2–4). The most frequently reported toxins in *Microcystis* blooms are microcystins. Microcystins are cyclic heptapeptides synthesized on large modular nonribosomal peptide synthetase (NRPS) and polyketide synthase (PKS) enzyme complexes (5). A large number of microcystin variants are synthesized due to cluster genetic diversity, particularly in the substrate-binding pockets of these enzymes (6). Indeed, these microcystin variants may differ in their toxicological effects. Therefore, a better understanding of the genetic structure of *M. aeruginosa* is relevant in light of toxin biosynthesis and control of toxic cyanobacterial blooms. To date, only the complete genome of *M. aeruginosa* strain NIES-843 isolated from a lake in Japan (7) and a draft of the strain PCC7806 that originated from a water reservoir in The Netherlands (8) have been published, but 17 more are underway (http://www.genomesonline.org).

SPC777 is the first strain of the species *M. aeruginosa* from South America to have its genome sequenced and it is also the first unicellular cyanobacterium reported as a saxitoxin producer (9). This strain was isolated from a water reservoir used for public supply (Riacho Grande branch of the Billings Reservoir, São Bernardo do Campo, SP, Brazil, 23°46′42.61′′S and 46°31′1.57′′W). The genome sequencing of SPC777 was carried out using the high-throughput platform combination of SOLiD V3, SOLiD 5500, Ion Torrent, and Illumina MiSeq. *De novo* genome assembly was carried out independently using CLC Genomics Workbench v. 6.0.2 (CLC Bio, Aarhus, Denmark) and Velvet sequence assembler v. 1.2.09 (10). The contig scaffolds were generated by use of the SIS program (11), using *M. aeruginosa* NIES-843 as the reference genome. The final genome assembly has 1,040-fold coverage and contains 278 contigs (>500-bp length) composing a total of 11 scaffolds with a total size of 5,457,256 bp and a mean GC content of 42.63%. The draft genome was annotated using Prokka v1.5.2 (http://www.vicbioinformatics.com/software.prokka.shtml), which predicted 5,234 coding sequences (CDS), whereas 3,024 CDSs have functional assignment. The draft metabolic models were reconstructed using the RAST annotation system (12). Preliminary genome analysis revealed that strain SPC777 carries gene clusters implicated in nonribosomal biosynthesis of microcystins, aeruginosin, and terpenes. Likewise, gene clusters involved in ribosome-dependent synthesis of peptides such as microcyclamide (cyanobactin), microviridin, microvirin, and bacteriocins were found, which have potential biotechnological applications. Additionally, the complete gene cluster involved in aerotope formation was identified. Two copies of the 16S rRNA gene were obtained from the annotation and both were 99% identical to those of *M. aeruginosa* NIES-843. Further detailed analysis, including functional annotation, comparative genomics, and metabolic pathways analysis at the genome scale is in process and the results will be included in our future publication.

### Nucleotide sequence accession numbers. 

The draft genome sequence of *Microcystis aeruginosa* SPC777 has been deposited at DDBJ/EMBL/GenBank under the accession number ASZQ00000000. The version described in this paper is version ASZQ01000000.
